# Action Video Gaming Enhances Brain Structure: Increased Cortical Thickness and White Matter Integrity in Occipital and Parietal Regions

**DOI:** 10.3390/brainsci15090956

**Published:** 2025-09-02

**Authors:** Chandrama Mukherjee, Kyle Cahill, Mukesh Dhamala

**Affiliations:** 1 Department of Physics and Astronomy, Georgia State University, Atlanta, GA 30303, USA; cmukherjee1@student.gsu.edu (C.M.); kcahill.neurophys@gmail.com (K.C.); 2 Neuroscience Institute, Georgia State University, Atlanta, GA 30303, USA; 3 Center for Behavioral Neuroscience, Center for Diagnostics and Therapeutics, Georgia State University, Atlanta, GA 30303, USA; 4 Tri-Institutional Center for Translational Research in Neuroimaging and Data Science (TReNDS), Georgia State University, Georgia Institute of Technology, and Emory University, Atlanta, GA 30303, USA

**Keywords:** video game playing, action video games, brain gray matter thickness, dMRI, parietal, occipital

## Abstract

Background: Action video games—particularly first-person-shooter (FPS), real-time-strategy (RTS), multiplayer-online-battle-arena (MOBA), and battle-royale (BR) titles—have been linked to enhanced visuospatial skills, yet their impact on brain structure remains unclear. Purpose: To examine, using a cross-sectional design, whether long-term exposure to high-speed genres is associated with variations in cortical thickness and white matter microstructure. Methods: Structural and diffusion MRI were acquired from 27 video-game players (VGPs) and 19 non-video-game players (NVGPs). FreeSurfer-derived cortical thickness and DSI-Studio quantitative anisotropy (QA) were compared between groups, co-varying for intracranial volume. All *p*-values were Holm–Bonferroni- and FDR-corrected; bootstrap 95% CIs are reported. Results: VGPs showed greater cortical thickness in right inferior and superior parietal, supramarginal, and precuneus cortices (η*_p_*^2^ = 0.12–0.21) and higher QA along right SOG–SPL and left SOG–IPL tracts. Conclusions: Frequent action gaming is associated with greater cortical thickness in the dorsal stream and enhanced occipito-parietal connectivity. However, causal inference is precluded; longitudinal work is warranted.

## 1. Introduction

Video games are now played by ~3.3 billion people worldwide [1], generating >USD 180 billion annually. While benefits ranging from improved attention to surgical precision have been documented, the structural correlates of prolonged play—especially for action genres that tax visuomotor circuits—remain incompletely understood. Video games have become an integral part of modern entertainment, with research showing that consistent gameplay can lead to noticeable changes in brain structure and function [2,3,4,5,6]. Studies have highlighted that video gaming can affect brain regions associated with visuospatial abilities, motor control, and attentional processes, suggesting that gaming experience may shape cognitive and neural development [7,8,9,10]. Structural neuroimaging studies have reported alterations in cortical thickness [11] and white matter integrity [12] in individuals who regularly play video games (VGPs), indicating that the brain undergoes adaptive modifications in response to the cognitive and sensorimotor demands of gaming activities. As a result, researchers are increasingly interested in exploring the long-term effects of video game exposure both beneficial and detrimental [3,13,14]. On the one hand, gaming has been linked to cognitive, emotional, motivational, and social benefits [12] including improvements in reaction time, decision-making, and attention control. Conversely, concerns have been raised about potential negative effects, including exposure to violent content, gaming addiction [15], obesity, and various cardio-metabolic health risks [16]. Systematic reviews of video gaming’s effects on the brain have highlighted structural and functional adaptations observed through MRI studies, with changes linked to enhanced neuroplasticity, improved cognitive functions, and superior visuospatial skills [3,13,17]. However, despite increasing evidence of neuroplastic adaptations, the extent to which different gaming genres influence brain structure remains uncertain.

One key structural change linked to the action video gaming experience is cortical thickness, a well-established marker of gray matter integrity and cognitive function [18]. Increased cortical thickness has been associated with enhanced neural efficiency, increased dendritic complexity, and reduced synaptic pruning, all of which may contribute to superior visuospatial and sensorimotor performance [11,17,19]. Given the high cognitive and perceptual demands of action video games, regions involved in spatial attention, decision-making, and motor coordination such as the parietal cortex may be particularly affected [2,11,19,20]. However, gray matter alone does not fully capture the structural changes in the brain associated with gaming. White matter connectivity plays a crucial role in facilitating communication between brain regions, with greater connectivity supporting more efficient cognitive processing and sensorimotor coordination [21].

Diffusion-based imaging techniques assess microstructural white matter properties, with quantitative anisotropy (QA) serving as a marker of axonal density and connectivity strength [22,23]. Despite fractional anisotropy (FA) being a more commonly reported measure of structural connectivity compared with QA, FA suffers more from confounds such as crossing fibers and free water effects, informing our decision to utilize QA as the primary measure of structural connectivity in our investigation. Since action video games require rapid visuomotor integration, it is essential to investigate whether changes in cortical thickness are accompanied by strengthened white matter connectivity along visuospatial processing pathways. These games predominantly engage the occipital and parietal regions within the dorsal stream—the “where pathway—which plays a crucial role in perception-action coupling [24,25]. This study examines structural brain changes associated with action video gaming, with a focus on cortical thickness and white matter integrity in the dorsal stream—a key network for visuospatial processing, perceptual decision-making, and motor planning.

## 2. Materials and Methods

### 2.1. Subject Data

The number of participants for the structural connectivity analysis was 46 total participants (27 video game players—gamers (20 ± 2 years of age), and 19 non-video game players, non-gamers (20 ± 2 years of age)). Participants were recruited by posting flyers physically and digitally on approved areas at Georgia State University Atlanta, Georgia, United States and neighboring universities also located in Atlanta, Georgia, United States areas, and by advertising on regional video game group Facebook pages. Participants were asked to complete a questionnaire about their video gaming habits to determine their group classification, following guidelines like those used in prior studies by Green and Bavelier [26,27,28] and others (e.g., Gao et al., 2018; Stewart et al., 2020) [29,30].

A participant was categorized as a video game player (VGP) if they reported playing video games for at least 5 h per week over the past year, consistent with the criteria set by Green and Bavelier, who used a similar threshold of 5 h per week over six months. Those who reported playing more than 5 h weekly in one of four specific game genres—first-person shooter (FPS), real-time strategy (RTS), multiplayer online battle arena (MOBA), or battle royale (BR)—during the previous two years were also considered VGPs. Non-video game players (NVGPs) who were recruited for this study played less than 30 min per week over the last two years prior to data collection. All participants passed the Ishihara test for color vision deficiency [31]. Before participation, they provided written informed consent and completed health screenings. Compensation was provided for their involvement, and the study received approval from the Institutional Review Boards of Georgia State University and Georgia Institute of Technology, located in Atlanta, Georgia.

### 2.2. Structural MRI Data

Whole-brain T1w and diffusion MR imaging was conducted on a 3 T Siemens Magnetom Prisma MRI scanner (Siemens, Atlanta, GA, USA) at the joint Georgia State University and Georgia Institute of Technology Center for Advanced Brain Imaging, Atlanta, GA, USA. High-resolution anatomical MR images were acquired for voxel-based morphometry and anatomical reference using a T1-MEMPRAGE scan sequence (TR = 2530 ms; TE1-4: 1.69–7.27 ms; TI = 1260 ms; flip angle = 7 deg; voxel size 1 mm × 1 mm × 1 mm) [9]. Diffusion-weighted imaging (DWI) data were acquired using a multi-shell diffusion scheme with b-values of 300, 650, 1000, and 2000 s/mm^2^, corresponding to 4, 17, 39, and 68 diffusion-encoding directions, respectively. One non-diffusion-weighted (b = 0) volume was also collected. The acquisition was performed using a single-shot echo-planar imaging (EPI) sequence with anterior-to-posterior (AP) phase encoding. Each diffusion volume consisted of 60 axial slices acquired with 2 mm isotropic resolution (slice thickness = 2 mm, in-plane resolution = 2 × 2 mm). The field of view (ReadoutFOV) was 220 mm, and the acquisition parameters were TR = 2750 ms, TE = 79 ms. The total scan duration was approximately 6.5 min.

### 2.3. Structural Data Analysis Pipeline

#### 2.3.1. Image Preprocessing

The anatomical data processing and morphometric parameter estimation were carried out using the ‘recon-all’ pipeline in FreeSurfer (ver. 7.4.1) available at https://surfer.nmr.mgh.harvard.edu/ (accessed on 14 November 2024) [32,33]. Processing was performed on a macOS system equipped with Apple Silicon (M2 Pro chip), 32 GB RAM, and integrated GPU acceleration.

Head motion artifacts were corrected using intensity normalization and rigid-body alignment to ensure that minor movements during scanning did not compromise the results [34]. Each brain was transformed into Talairach space and underwent cortical and subcortical segmentation based on the Desikan–Killiany atlas [35]. Additional details can be found in earlier studies [32,36,37,38].

#### 2.3.2. Data Extraction and Preparation

Subject-specific cortical thickness (CT) for bilateral cortical regions and intracranial volume (ICV, representing head size) were assessed using the mri_surf2surf, mris_anatomical_stats, and aparcstats2table tools, as part of the FreeSurfer recon-all pipeline [36]. The whole brain was parcellated into 70 cortical regions (35 in each hemisphere) based on Desikan’s atlas [35].

Quality control was rigorously performed by visually inspecting raw structural images, skull-stripped volumes, and reconstructed pial surfaces within FreeSurfer’s visual tools (e.g., Freeview). Criteria for quality control included accurate skull stripping without visible dura, consistent cortical ribbon delineation, and correct anatomical segmentation. Manual interventions such as control point edits, brain mask adjustments, and pial surface regeneration commands (recon-all-all-subjid subjectID) were used to correct identified segmentation and normalization errors [39].

#### 2.3.3. Statistical Analysis

Statistical analysis was conducted using IBM SPSS version 29.0 (IBM Corp., Armonk, NY, USA). A multivariate analysis of covariance (MANCOVA) was performed to examine the effects of the independent variable (group) on the dependent variables (thickness of 70 ROIs in the brain), with total intracranial volume (ICV) included as a covariate. Main effects and interactions were assessed, with statistical significance set at *p* ≤ 0.05, and effect sizes were reported using F-values and partial eta squared (η*_p_*^2^) values. Given our group sizes (27 vs. 19), the analysis is powered primarily to detect medium-to-large effects; smaller effects may be missed after correction.

### 2.4. Regions of Interest Comprising the Dorsal Stream

Our study focuses on the dorsal stream because these regions are directly involved in the processing and integrating of visuomotor information, which is central to our task involving moving visual stimuli [40,41]. Thus, our choice of ROIs reflects the specific neural mechanisms underpinning the task we investigated, rather than those associated with higher-order, reward-based decision-making. In our investigation, we examined the structural and functional organization of the dorsal streams using regions of interest (ROIs) defined in a prior study that found dorsal stream ROIs from the Neurosynth functional meta-analysis platform (https://neurosynth.org/) with MNI coordinates denoted in Table 1 [42]. The identified ROIs comprising the dorsal stream encompassed six regions, including the bilateral inferior parietal lobule (IPL) and superior parietal lobule (SPL). The nomenclature used in this classification was based on the Eickhoff–Zilles macro-labels from N27 and was implemented in AFNI [42]. The pairwise links between these regions follow a standard path for the dorsal stream: SOG—IPL and SOG—SPL. We employed the MNI coordinate system and constructed the ROIs using the FSLeyes visualization tool within the FSL version 6.0.6 (FMRIB Software Library) environment. Statistical comparison of connectivity metrics between ROIs across gamers and non-gamers was carried out using the Wilcoxon rank-sum test (also known as the Mann–Whitney U test). This nonparametric test was chosen because it does not assume the normality of the connectivity metrics’ distribution, making it suitable for comparing the two groups without requiring any assumptions about the underlying distribution of the data. For multiple comparison corrections in this analysis, we employed the Holm–Bonferroni method [43,44]. This method was selected for its ability to enhance statistical power and sensitivity to individual significant comparisons while effectively controlling for Type I errors. We applied a significance threshold of *p* < 0.05, with the Holm–Bonferroni correction indicated by p*, to ensure statistical significance while simultaneously controlling for the family-wise error rate in the dorsal stream, providing a robust framework for identifying meaningful differences in connectivity metrics.

### 2.5. Tractography Protocols

DSI Studio version 2022.08.0 is a non-commercial software program that was utilized in this study for diffusion MR image analysis and provided functions including deterministic fiber tracking and 3D visualization [22]. We used a multi-shell diffusion scheme with b-values of 300, 650, 1000, and 2000 s/mm^2^. The acquisition parameters consisted of an in-plane resolution of 2 mm and a slice thickness of 2 mm. The accuracy of b-table orientation was examined by comparing fiber orientations with those of a population-averaged template [45]. Diffusion-weighted images were preprocessed using the EDDY tool implemented via DSI Studio, which corrected for eddy current-induced distortions and motion artifacts. Reverse phase-encoding images were not acquired; therefore, susceptibility distortion correction using TOPUP was not applicable. The tensor metrics were then calculated.

The diffusion data were reconstructed in the MNI space using q-space diffeomorphic reconstruction [23] to obtain the spin distribution function [46]. A diffusion sampling length ratio of 1.25 was used. The resulting diffeomorphic reconstruction output had an isotopic resolution of 2 mm. The accuracy of b-table orientation was examined by comparing fiber orientations with those of a population-averaged template [45]. Diffusion-weighted images were preprocessed using the EDDY tool implemented via DSI Studio, which corrected for eddy current–induced distortions and motion artifacts. Reverse phase-encoding images were not acquired; therefore, susceptibility distortion correction using TOPUP was not applicable. The tensor metrics were then calculated. The diffusion data were reconstructed in the MNI space using q-space diffeomorphic reconstruction [23] to obtain the spin distribution function [46]. A diffusion sampling length ratio of 1.25 was used. The resulting diffeomorphic reconstruction output had an isotopic resolution of 2 mm.

Seeds were randomly placed throughout the ROIs until reaching a cutoff at 50,000,000 seeds. Additionally, two pairwise spherical ROIs were also defined as ending regions. In the case between the Left Superior Occipital Gyrus (L SOG) and the Left Inferior Parietal Lobule (L IPL), for example, the ending regions were placed at (52,74,37) and (51,64,51). An angular threshold of 60 degrees was set as the maximum allowed angular deviation between steps. The step size was randomly selected from 0.5 voxels to 1.5 voxels.

Tracks with lengths shorter than 10 mm or longer than 100 mm were excluded from further analysis. The process continued until mapping of the dorsalstreams, with an exhaustive exploration of all pairwise links in each section denoted in Table 2. For full reproducibility, the parameter ID used in DSI Studio to configure the settings described above is provided: 0AD7233C9A99193Fba3Fdb2041bC84280F0FA02ec. This ID allows others to load the exact parameters used in our analysis, ensuring that tractography and derived metrics can be replicated using identical configurations. Parameter IDs for each pairwise link are available on the Open Science Framework (OSF) repository accompanying this project.

## 3. Results

### 3.1. Cortical Thickness

**Figure 1:**
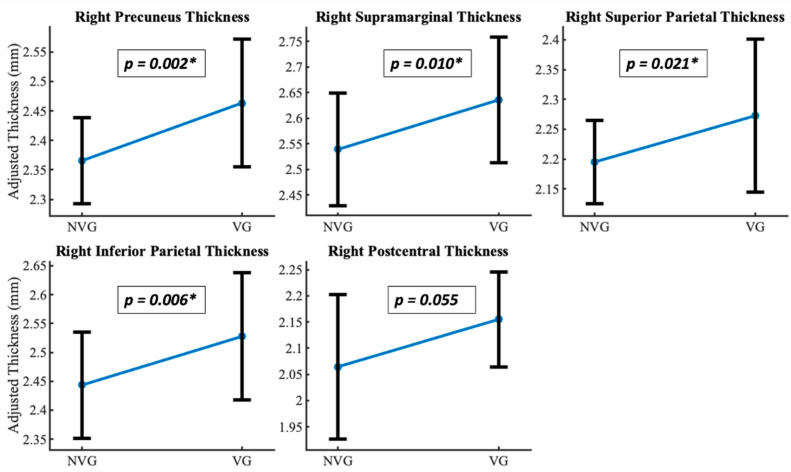
Gray matter thickness differences. Non-video game players (NVG) and video game players (VG) differed significantly (corrected *p* < 0.05) in gray matter thickness in parietal and occipital areas: Right precuneus, right supramarginal, right superior parietal, right inferior parietal, and right postcentral. (*) denotes the significant *p* values for the regions, respectively.

**Figure 2:**
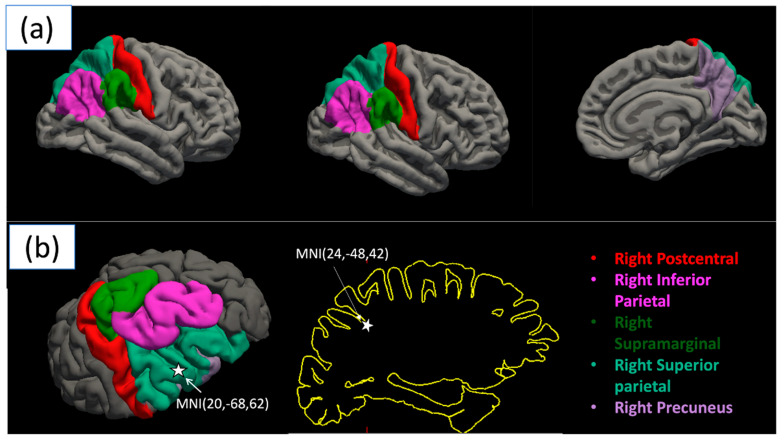
Regions of significant gray matter thickness differences between video gamers and non-gamers. (**a**) shows the statistically significant regions, whereas (**b**) shows the MNI coordinates along the right superior parietal and right inferior parietal areas, which are a part of the dorsal visual processing stream.

A significant difference in cortical thickness was observed between groups [F(2,41) = 19.828, *p* = 0.049, η*_p_*^2^ = 0.998]. The most significant regions are shown in Table 3. The results depict a significant difference in cortical thickness between video game players (VGP) and non-video game players (NVGP), with all reported *p*-values Bonferroni-corrected for multiple comparisons across 70 ROIs to control for Type I error. The four significant regions, namely right inferior parietal, right precuneus, right superior parietal, and right supramarginal, show the most significance among those identified, as shown in Figure 1 and Figure 2a. We also found the MNI coordinates along the right superior parietal and right inferior parietal areas, which are a part of the dorsal visual processing stream, as shown in Figure 2b.

### 3.2. White Matter Integrity

**Figure 3:**
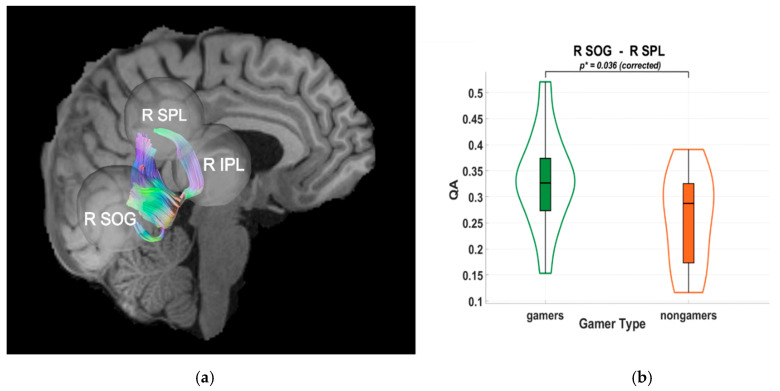
(**a**) Tractography visualization in a representative subject showing white matter connections between the right superior occipital gyrus (R SOG), right superior parietal lobule (R SPL), and right inferior parietal lobule (R IPL). The x-axis is coded for red from right to left, the y-axis is coded for green from anterior to posterior, and the z-axis is coded for blue from superior to inferior. (**b**) Violin plot of group differences in quantitative anisotropy (QA) between video game players (VGPs; “gamers”) shown in green and non-video game players (NVGPs; “nongamers”) shown in orange for the R SOG–R SPL connection. Frames and grid lines denote the standard axes of the plot: the y-axis shows QA values, while the x-axis separates the two groups. Significantly higher QA values were observed in VGPs compared to NVGPs (p* = 0.036, Holm-Bonferroni corrected).

**Figure 4:**
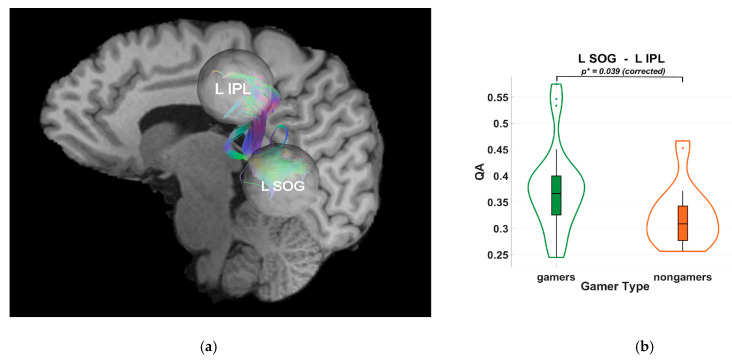
(**a**) Tractography visualization in a representative subject showing white matter connections between the right superior occipital gyrus (R SOG), right superior parietal lobule (R SPL), and right inferior parietal lobule (R IPL). The x-axis is coded for red from right to left, the y-axis is coded for green from anterior to posterior, and the z-axis is coded for blue from superior to inferior. (**b**) Violin plot of group differences in quantitative anisotropy (QA) between video game players (VGPs; “gamers”) shown in green and non-video game players (NVGPs; “nongamers”) shown in orange for the L SOG–L IPL connection. Frames and grid lines denote the standard axes of the plot: the y-axis shows QA values, while the x-axis separates the two groups. Significantly higher QA values were observed in VGPs compared to NVGPs (p* = 0.036, Holm-Bonferroni corrected).

Diffusion tractography analyses indicated higher quantitative anisotropy (QA) values in VGPs within dorsal-stream pathways. Specifically, significant group differences were observed between the right superior occipital gyrus (SOG) and the right superior parietal lobule (SPL) (*p* = 0.036), and between the left SOG and the left inferior parietal lobule (IPL) (*p* = 0.039), as shown in Figure 3 and Figure 4. These results were Holm–Bonferroni-corrected to control for multiple comparisons. The observed QA differences suggest an association between habitual gaming and microstructural characteristics of occipito-parietal white matter pathways, without implying causality.

## 4. Discussion

Our findings indicate that habitual action video game playing is associated with structural differences in key visuospatial processing and attention control networks in the right parietal cortex. Compared with non-video game players, VGPs showed greater cortical thickness in the right inferior parietal lobule (IPL), right superior parietal lobule (SPL), right precuneus, and right supramarginal gyrus—regions implicated in spatial attention, decision-making, and sensorimotor integration [47,48,49,50]. In addition, white matter connectivity analyses using Q-space tractography revealed higher quantitative anisotropy (QA) in two dorsal-stream pathways: right superior occipital gyrus (SOG)–right SPL and left SOG–left IPL. These patterns suggest a close correspondence between parietal cortical morphology and occipito-parietal structural connectivity in individuals with extensive action game experience. These structural brain changes help explain the behavioral findings previously reported by Jordan and Dhamala [8,9].

The SPL, a central hub for visuomotor coordination, showed greater cortical thickness in VGPs, consistent with prior reports of parietal morphological differences in experienced gamers. This region integrates visual, proprioceptive, and motor inputs to support rapid decision-making and goal-directed actions—skills frequently engaged during action game play [51]. Likewise, the IPL, which contributes to attentional control, sensorimotor integration, and action planning, displayed greater cortical thickness. Previous studies have shown that the IPL dynamically reallocates attention in unpredictable environments, a cognitive demand typical of fast-paced games. The precuneus, which also showed greater thickness, supports mental imagery, spatial reasoning, and self-referential processing, and is frequently activated in expert gamers during spatial tasks. Together, these associations are compatible with—but do not prove—a functional link between extensive gaming and visuospatial network morphology [48,52]. Increased cortical thickness in this region may reflect enhanced spatial navigation abilities and strategic planning, which are fundamental components of action video gameplay. Studies have shown that expert gamers exhibit greater activation of the precuneus during spatial tasks, supporting the idea that gaming strengthens this region’s role in complex visuospatial processing. The right-lateralized parietal thickening detected here dovetails with that behavioral profile, suggesting a plausible anatomical substrate for far-transfer gains. Longitudinal designs that pair structural imaging with rigorous cognitive batteries (pre-, mid- and post-training) will be essential to establish temporal precedence and dose–response relations. Older adults and patients recovering from stroke or traumatic brain injury often exhibit dorsal-stream deficits that impair navigation and visuomotor control. Pilot trials show that game-based interventions—especially when delivered in virtual or augmented reality—can boost adherence and enhance rehabilitation outcomes [53,54].

Our findings reveal an association between self-reported video game history and variations in cortical thickness and white matter microstructure, but they do not establish causality or directionality. Because group membership was determined by self-report, the observed structural differences could reflect pre-existing neuroanatomical characteristics, lifestyle factors, engagement in other visuospatially demanding activities, or socio-demographic influences. However, given that both cohorts were recruited from similar socio-demographic backgrounds, these alternative explanations are less likely. Longitudinal studies that integrate structural and functional neuroimaging with cognitive assessments will be essential to clarify temporal relationships and potential dose–response effects.

Our findings suggest that video games, particularly action games, could be leveraged as cognitive training tools for individuals with visuospatial processing and attentional deficits. Additionally, not all video games are likely to produce the same effects. Action games, which require rapid visuospatial processing and sensorimotor coordination, may drive different neural adaptations compared to puzzle or strategy games, which likely engage executive functions and problem-solving networks. Future research should explore how different genres and subgenres influence structural connectivity, helping to refine game-based cognitive training paradigms.

Finally, while our study focused on cortical thickness and white matter integrity, future research should examine how these structural adaptations interact with functional connectivity and real-time neural dynamics. Multimodal approaches, including resting-state and task-based fMRI, could help clarify how structural changes translate into behavioral advantages. Moreover, EEG studies demonstrate that video game play induces distinct attentional and arousal-related neural signatures, suggesting that future work should explore how these structural adaptations correspond to real-time cognitive processing efficiency [30].

Interpretation of these findings should be made in light of several methodological and conceptual limitations. First, our study focused on structural MRI measures (cortical thickness and diffusion-based quantitative anisotropy) without incorporating functional imaging or behavioral assessments. While these structural brain differences may help explain the behavioral and functional distinctions between VGPs and NVGPs previously reported by Jordan and Dhamala [8,9], we cannot determine whether they translate into altered network activity or enhanced cognitive performance in gamers. Moreover, despite efforts to ensure high-quality analyses, tractography is inherently limited by spatial resolution constraints, susceptibility to technical artifacts, and the modest sample size of our study. Second, we cannot conclude that action gameplay caused the greater parietal cortical thickness or white matter QA; individuals with certain neuroanatomical characteristics may simply be more inclined to engage in gaming (a selection effect), or unmeasured variables may account for the group differences. By deliberately avoiding causal terms such as ‘neuroplastic changes’ or ‘effects,’ we emphasize that the observed structural differences are associative findings only. Longitudinal training studies or pre–post designs will be required to establish directionality and causation.

Third, our modest sample size (27 VGPs vs. 19 NVGPs) and multiple statistical comparisons place some limits on statistical power and precision. Although we applied Holm–Bonferroni and FDR corrections to control for Type I error, studies of this scale are typically less sensitive to smaller effects. Thus, while our study was likely well powered to detect relatively large group differences, more subtle effects may have gone undetected. Future studies with larger cohorts will be important to confirm the robustness of these findings and to uncover smaller, yet potentially meaningful, brain differences.

## 5. Conclusions

Our results indicate that long-term action video game play is associated with distinctive structural characteristics in visuospatial and sensorimotor networks, particularly within the dorsal stream and right parietal cortex. These patterns suggest that gaming experience may be linked to differences in cortical and white matter organization that could support efficient visuospatial processing and attentional control. Further studies using training-based designs and longitudinal brain imaging are needed to clarify the underlying mechanisms and potential long-term implications. Nonetheless, this work positions gaming as a useful framework for investigating experience-related variation in brain structure and for developing targeted cognitive training strategies.

## Figures and Tables

**Table 1:** Dorsal stream regions for tractography.

Region of Interest	MNI Coordinates x, y, z (mm)
Left superior occipital gyrus (L SOG)	−26, −73, 23
Left inferior parietal lobule (L IPL)	−24, −52, 52
Left superior parietal lobule (L SPL)	−30, −46, 66
Right superior occipital gyrus (R SOG)	23, −91, 26
Right inferior parietal lobule (R IPL)	24, −48, 42
Right superior parietal lobule (R SPL)	20, −68, 62

**Table 2:** White matter pathways comprising the dorsal stream.

Connection	Min Length (mm)	Max Length (mm)	Angular Threshold (deg)
L SOG L IPL	10	100	60
L SOG L SPL	10	300	70
R SOG R IPL	10	100	75
R SOG R SPL	10	150	70

**Table 3:** Largest cortical thickness differences between VGPs and NVGP.Partial ETA squared(***η_p_*^2^**).

Identified Regions (Thickness)	*p*	*η_p_^2^*	F(1,42)
Right inferior parietal	0.006	0.169	8.521
Right precuneus	0.002	0.210	11.154
Right postcentral	0.055	0.085	3.902
Right superior parietal	0.021	0.121	5.779
Right supramarginal	0.010	0.148	7.293

## Data Availability

The data to generate the key findings of the study, as well as the scripts, can be found in OSF (https://osf.io/uz2n5/). The raw data can be made available upon request.
